# Context‐Dependent Effects of Amino Acid Supplementation on Nestling Growth and Baseline Innate Immune Function

**DOI:** 10.1002/ece3.73284

**Published:** 2026-03-18

**Authors:** Ashetu Debelo Terefa, Arne Hegemann, Zoltán Németh, Ádám Z. Lendvai

**Affiliations:** ^1^ Department of Evolutionary Zoology and Human Biology Debrecen University Debrecen Hungary; ^2^ Juhász‐Nagy Pál Doctoral School of Biology and Environmental Sciences Debrecen University Debrecen Hungary; ^3^ Department of Biology Ambo University Ambo Ethiopia; ^4^ Department of Biology Lund University Lund Sweden; ^5^ Institute of International Animal Health/One Health, Friedrich‐Loeffler‐Institut Federal Research Institute for Animal Health Greifswald ‐ Insel Riems Germany

**Keywords:** chicks, great tit, innate immunity, leucine, methionine, urbanization

## Abstract

Growth and immune development—fundamental parts of any organism's ontogeny—depend critically on nutrient availability, particularly on essential amino acids that support protein synthesis and physiological processes. However, environmental variation can reduce prey diversity and nutrient quality, producing suboptimal diets that lack essential amino acids, potentially constraining development. In this study, we experimentally supplemented great tit ( *Parus major* ) nestlings from forest and suburban environments with methionine, leucine, or tap water (control) from day 4 to day 7 post‐hatching and investigated the effects on growth and baseline innate immune function across each habitat. We assessed nestlings' growth using body mass, tarsus length, and wing length and baseline innate immune function by quantifying complement activity (measured as lysis) and natural antibody titers (measured as agglutination). Supplementation significantly increased body mass gain in forest nestlings, particularly in smaller individuals, suggesting enhanced protein synthesis efficiency, although its effects on tarsus and wing length were limited. Methionine significantly improved lysis activity, suggesting enhanced innate immune function, whereas agglutination was not notably affected. In contrast, suburban nestlings showed limited responses to supplementation, suggesting broader nutritional constraints beyond individual amino acid deficiencies. Habitat and initial body mass significantly influenced growth rate, but their effects depended on the treatment. These findings highlight the complex, condition‐dependent effects of amino acid supplementation on nestling development and emphasize the importance of considering habitat‐specific nutritional limitations when assessing avian developmental plasticity.

## Graphical abstract

We experimentally supplemented great tit nestlings with methionine, leucine, or tap water to test how essential amino acids influence growth and immune function in forest and suburban habitats. Supplementation increased mass gain in smaller forest nestlings and enhanced lysis activity with methionine in suburban populations. These results demonstrate context‐ and condition‐dependent nutritional effects and highlight habitat‐specific constraints on avian development.

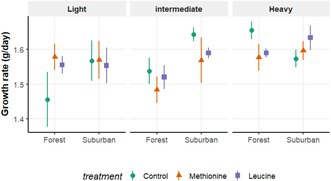

## 1 Introduction

In developing animals, resources must be allocated simultaneously to multiple functions—particularly growth and development of physiological systems, including vital immune defenses—and this allocation is strongly influenced by nutrient availability and quality (Zera and Harshman 2001). In birds, rapid growth and immune system maturation are especially critical, as nestlings must reach adequate body condition and immunocompetence within a short developmental window. For instance, body size at fledging often predicts survival, with fledglings possessing shorter wings experiencing increased mortality (Aastrup et al. 2023; Gerritsma et al. 2022; Jones et al. 2017; Jones and Ward 2020; Martin et al. 2014; Martin 2015; Morrison et al. 2009), and nestlings with weaker immune function may be more susceptible to infection (Merino et al. 2000). Among nutrients, amino acids play a central role in supporting these developmental processes (Alagawany et al. 2021; Herring et al. 2021; Liu et al. 2022; Ndunguru et al. 2024a, 2024b). Amino acids, as the building blocks of proteins, are essential to support tissue development, regulate enzymatic and metabolic functions, and modulate the immune defenses as well as the maintenance of physiological homeostasis (Alagawany et al. 2021). As such, insufficient amino acid levels can impair growth, health and compromise body composition, whereas excessive intake of certain amino acids can be toxic or lead to metabolic imbalances (Swennen et al. 2007).

Amino acid requirements vary substantially depending on diet type and prey composition (Langlois and McWilliams 2022; Nicolson 2007; Wendeln et al. 2000). Food items differ markedly in their amino acid content: for example, fruits and many plant‐based foods are relatively poor or imbalanced in essential amino acids, whereas arthropods commonly consumed by birds are generally rich in protein and essential amino acids (like methionine, lysine, and tryptophan), although their relative composition varies among taxa (Langlois and McWilliams 2022; Reeves et al. 2023). Insectivorous birds therefore heavily rely on arthropods, which can meet avian nutritional demands even when consumed in relatively small amounts (Finke 2015; Ramsay and Houston 2003). Among arthropod prey, caterpillars represent the primary food source for many altricial passerine nestlings during early development (Cowie and Hinsley 1988; Höhn et al. 2024; Ramsay and Houston 2003; Wawrzyniak et al. 2020). Spiders provide complementary essential amino acids and are particularly important during the first days after hatching, with peak parental provisioning occurring around nestling days 5–6 (Cowie and Hinsley 1988; Pagani‐Núñez et al. 2011; Ramsay and Houston 2003).

Variation in prey availability across habitats, particularly between forest and urban environments, can shape parental provisioning strategies and contribute to differences in nestling development (Mägi et al. 2009; Höhn et al. 2024). Abundance of high‐quality prey, such as caterpillars associated with oak‐dominated habitats, has been linked to increased nestling body mass and wing length (Jensen et al. 2023). In contrast, urbanization is often associated with reduced nestling size and mass, although these effects may be most pronounced at highly urbanized sites (Biard et al. 2017; Seress et al. 2020). Studies of blue tits further show that urban parents travel longer distances compared to rural conspecifics to provision their young yet still deliver diets of lower nutritional quality, characterized by a reduced proportion of caterpillars, resulting in lower nestling mass and reproductive output than blue tits from rural areas (Jarrett et al. 2020; Serrano‐Davies and Sanz 2017). More generally, reductions or change in prey availability whether because of lower biomass or decreased nutritional quality can consistently lead to slower mass gain during critical stages of nestling development, while also affecting immune function and overall physiological condition (Greenberg 1995; Johnson et al. 2005; Lacombe et al. 1994; Mägi et al. 2009; Senécal et al. 2021).

Among the nine essential amino acids, methionine and leucine are particularly important for promoting early development in nestlings. Methionine plays a key role in protein synthesis, cellular metabolism, and is a key precursor of glutathione, a major antioxidant that protects cells from oxidative stress (Grimble and Grimble 1998; Langlois and McWilliams 2022; Liu et al. 2022). Methionine is also involved in methylation reactions that regulate gene expression, and limited methionine intake can reduce immune responses and impair growth (Fagundes et al. 2020). In many birds, methionine is often the first limiting amino acid, and its scarcity reduces growth rates in nestlings and negatively affects reproductive traits, such as egg size and production in adults (Keshavarz 2003; Sekiz et al. 1975). Furthermore, shortages in methionine can lead to trade‐offs between growth and immunity (Brommer 2004; Mirzaaghatabar et al. 2011; Soler et al. 2003; Zhang et al. 2024). Leucine, a branched‐chain amino acid, promotes growth by stimulating muscle protein synthesis via the mTOR signaling pathway and supports translation of mRNA into proteins ensuring efficient nutrient utilization during rapid growth phases (Li et al. 2009; Nagao et al. 2015; Palii et al. 2009; Wu 2013). Leucine also has immunomodulatory functions and may reduce protein breakdown during periods of stress or nutritional deficiency (Bonvini et al. 2018). Leucine supplementation has been shown to promote muscle growth, improve feed efficiency and modulate the immune response in domestic birds (Izumi et al. 2004; Xie et al. 2019). However, despite their known importance, the effects of methionine and leucine availability on growth and immune function in free‐living bird species remain underexplored.

Supplementation studies in wild birds offer a promising approach to investigate nutritional limitations and potential trade‐offs between growth and immune function. Most previous studies have focused on general food supplementation, such as providing mealworms or caterpillars (e.g., Seress et al. 2020), or the effects of carotenoids or antioxidants (Biard et al. 2006; Tschirren et al. 2005), but only a few have manipulated specific amino acid intake. In this context, Tschirren et al. (2005) and Wegmann et al. (2015) found reduced or no effect of methionine supplementation on growth in great tit ( *Parus major* ) nestlings, but reported enhanced T‐cell‐mediated immune competence. Similarly, studies on other species, such as blue tits ( *Cyanistes caeruleus* ) and magpies ( *Pica pica* ), have shown that methionine supplementation can improve immunocompetence, although often at the cost of reduced growth (Brommer 2004; Soler et al. 2003).

In this study, we experimentally tested the effects of methionine and leucine supplementation on nestling growth and on components of baseline innate immune function in great tits. Innate immunity constitutes the first line of defense against pathogens, providing rapid, non‐specific protection prior to the activation of adaptive immune responses. We quantified natural antibodies and complement activity, which are widely used indicators of baseline innate immunity in avian ecological studies (Buehler et al. 2011; Matson et al. 2006, 2005).

We hypothesized that methionine and leucine supplementation would influence nestling growth and innate immune function by altering resource allocation between competing physiological demands. Given their essential roles in protein synthesis, metabolism, and immune regulation, we predicted that these amino acids may act as limiting resources during early development. On the basis of previous studies, we further expected that supplementation could mediate physiological trade‐offs between somatic growth and immunocompetence (Brommer 2004; Soler et al. 2003; Tschirren and Richner 2006). For example, leucine is known to promote muscle growth, whereas methionine can enhance innate immune function, potentially at the cost of growth (Izumi et al. 2004; Xie et al. 2019). If any of these nutrients are limiting in the natural diet of nestlings, supplementation can make a measurable difference, which could be enhancing or constraining growth and immunity simultaneously. We additionally considered the possibility that responses to supplementation may be context‐dependent and influenced by environmental factors that affect diet quality, nestlings' initial body mass, and nestling age.

## 2 Materials and Methods

### 2.1 Field and Experimental Treatment Protocol

We monitored breeding of great tits in 218 nest boxes installed in three clusters in Debrecen, Hungary, between March and June 2022. Nest boxes were located in (1) a contiguous oak‐dominated forest (*n* = 72; centroid: 47.5607, 21.6194), in (2) the Botanical Garden, a large, semi‐managed area with high canopy cover and limited human disturbance (*n* = 102; centroid: 47.5577, 21.6199), and in (3) open urban parks interspersed with buildings, pedestrian and vehicle traffic within the campus of University of Debrecen (*n* = 54; centroid: 47.5552, 21.6218). On the basis of vegetation structure, degree of habitat fragmentation, and human activity, we categorized the forest and Botanical Garden as ‘forest’ habitats, and the university parks as a ‘suburban’ habitat.

Starting in the second week of March, nest boxes were checked twice a week for signs of nest building. To determine the precise dates of egg‐laying and hatching, occupied nest boxes were visited three times a week from the date of occupancy onwards, whereas empty nest boxes were checked once a week until they became occupied or till the end of the breeding season. Once final clutch size was reached, we paused visits for 10 consecutive days to avoid disturbance and minimize risk of nest abandonment. Two days before the anticipated hatching date (i.e., 10 days after a clutch was complete), we checked the nest boxes once per 24 h to determine the hatching date (day 0).

On day 4 post‐hatching, we individually marked all nestlings. Although we primarily used metal rings for identification, for nestlings too small to accommodate a ring (*n* = 17), we applied temporary color markings (violet, red, green, and orange) on their head until they reached a size when they could be ringed with a numbered metal ring. At the day of first marking, that is, day 4‐post‐hatching, we also weighed all nestlings (*n* = 168 in 28 broods) to the nearest 0.01 g using a portable electronic balance. On the basis of their body mass, we ranked and grouped them into blocks of three, starting with the three heaviest nestlings. Using a randomized block design, we assigned each of the three nestlings in the first block randomly to one of the three amino acid supplementation treatments (methionine, leucine, and control; see below). All broods contained at least one block, and for broods that contained more than five nestlings, we reversed the order of treatment in the subsequent block. This procedure was repeated until all nestlings were assigned to a treatment group. In total, we formed 73 blocks: 26 first, 26 second, 18 third, and 3 fourth blocks.

### 2.2 Amino Acid Solutions and Oral Administration of the Treatments

We prepared a leucine solution (19 mg/mL) by mixing L‐leucine powder (CAS No. 61‐90‐5, Prod. No. W329703, purity: **≥** 99%, Sigma Aldrich) and a methionine solution (30 mg/mL) by mixing L‐methionine powder (CAS No. 63‐68‐3, prod. No. 64319, BioUltra > 99.5%, Sigma Aldrich) with distilled water. The solution's concentration was close to each amino acid's respective maximum solubility in water. The methionine and leucine treatment groups received 100 μL of each solution, respectively, and the same volume of tap water was substituted for the control group. An automated pipette was used to administer the solutions (methionine, leucine, distilled water) orally to all nestlings within the respective treatment groups. Nestlings received the amino acid solutions or the water once daily between 6:00 and 17:00 from day 4 to day 7 of age.

### 2.3 Measurements of Growth and Blood Sampling

After each supplementation (see above), we recorded the body mass of each nestling to the nearest 0.01 g using an electronic balance. On day four (i.e., first day of the supplementation) and on day 8 (i.e., 1 day after the last supplementation), we also measured tarsus and wing length using calipers (±0.1 mm) and rulers (±1 mm), respectively. Blood samples were collected on day 8 from the brachial wing vein of nestlings using capillary tubes and temporarily stored on ice in the field. We used sterile 17G hypodermic needles for blood sampling. Returning to the lab the same day, blood samples were centrifuged at 1431 RCF for 5 min. Plasma and red blood cells were separated and stored at −20°C until subsequent laboratory analysis.

### 2.4 Hemagglutination and Haemolysis Assay

To assess natural antibody and complement activity in nestling plasma, we used a hemagglutination and hemolysis assay as per Matson et al. (2005). We chose this assay as natural antibody titers and complement activity have been shown to develop during the nestling phase (e.g., Aastrup and Hegemann 2021; Killpack et al. 2013; Mauck et al. 2005), are sensitive to environmental and individual conditions of the nestlings (Aastrup et al. 2023; Nwaogu et al. 2023; Wemer et al. 2021) and have predictive capacity of survival (Hegemann et al. 2015; Roast et al. 2020). Because of the limited plasma volume available, we could not do any additional immunological assays. We centrifuged fresh rabbit blood collected from domestic rabbits from the Department of Pharmacology and Pharmacotherapy, Debrecen University, and stored with EDTA at 1431 RCF for 5 min to separate cellular components from the supernatant. We then measured the hematocrit value of the RBCs and used it to prepare a 1% RBC working solution in a phosphate‐buffered saline (PBS) for the assay. Phosphate‐buffered saline was prepared in‐house using standard laboratory reagents following a conventional PBS formulation.

The assay was conducted on 96‐well, round‐bottom microplates (Cat. No. 4912, Thermo Fisher Scientific). Control wells were established by adding 12.5 μL of PBS as negative controls and 12.5 μL of pooled Japanese quail ( *Coturnix japonica* ) plasma as positive controls. Plasma samples (12.5 μL each) of great tit nestlings were loaded into a plate, and a serial dilution was performed by sequentially aspirating, mixing, and transferring 12.5 μL across columns using a multichannel pipette. Finally, 12.5 μL of 1% rabbit RBC suspension was added to each well. Plates were sealed, incubated at 37°C for 90 min, dried at a 45° angle for 20 min, and photographed for agglutination scoring. After another 70 min of horizontal drying, the plates were photographed for lysis scoring. Scoring was done following Matson et al. (2005) and blindly with respect to treatment category. The photos were randomized to allow for blind scoring with respect to sample ID. The strips were scored twice on different days by the same person (ADF). We calculated the final score as the mean of these two scores. If the scores differed by more than one, the same person scored the strip a third time, and the final measurement was calculated as the median of the three scores (cf. Ventura et al. 2025; Klumpp and Hegemann 2026).

### 2.5 Data Analysis

We analyzed the effects of amino acid supplementation on nestling growth and immune function in linear mixed‐effects models using R (version 2023‐10‐31). We analyzed both the repeated raw measurements (tarsus, wing length, and body mass) and the growth rates (delta values). This allowed us to test the existence of any pre‐treatment (day 4) differences in the measured traits, while simultaneously analyzing treatment effects and the influence of age, environment, and developmental conditions. To calculate growth rates (delta values), we subtracted the post‐treatment values (day 8) from the pre‐treatment values (day 4) for tarsus and wing length. For body mass, we used slope estimates from best linear unbiased predictions (BLUPs) as growth rates (delta values).

To analyze the effect of amino acid supplementation on body mass, we used linear mixed models using the lmer function from the lmerTest package (Kuznetsova et al. 2017) using raw repeated data as the response variable, and treatment, age, initial body mass, and habitat type as fixed effects. We also included all possible interactions. Nest box ID and individual ID (nested within nest box ID) were included as random effects. Hatching date and clutch size were tested but excluded from the final models because of their lack of significance. Also, to analyze the effect of amino acid supplementation on growth rate (delta values), we used linear mixed models and included treatment, initial body mass, and habitat type as fixed effects, as well as all possible interactions. Individual ID nested within nest box ID was included as a random effect.

Hemagglutination was evaluated using a linear mixed effects model, whereas hemolysis was analyzed using generalized linear mixed models with negative binomial distribution (Brooks et al. 2017). These different statistical models were used because of variation in the dataset distribution. However, for both response variables, we used the same model structure as for the delta values, that is, treatment, initial body mass, and habitat type were included as fixed effects with all their interactions, whereas nest ID was specified as a random effect in both models.

**FIGURE 1:**
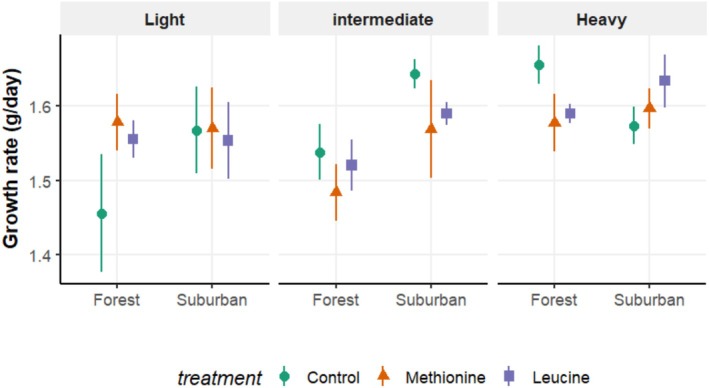
Best linear unbiased prediction (BLUP) slopes of body mass growth (delta values) in Great Tit nestlings across habitats (forest vs suburban) as a function of initial body mass. The BLUP slopes were derived from a linear mixed‐effects model including all fixed effects and their interactions listed in Table 2. Great tit nestlings received either methionine, leucine, or tap water (control group) through oral supplementation and were categorized into three groups: Light, intermediate, and heavy (for illustration purposes) on the basis of their initial (day 4) body mass measurement. Note that in the statistical model, initial body mass was treated as a continuous covariate, and the categorization into three classes is done for visualization only (see statistical methods).

Initial body mass was included as a continuous variable in the analyses (see above) to assess its potential as a predictor of growth rate (Ronget et al. 2018). However, for better visualization, we categorized nestlings into three phenotypic classes: light, intermediate, and heavy on the basis of their initial body mass measured on day 4, as shown in Figure 1. This categorical classification facilitated an easier visualization of condition‐dependent treatment effects and was not used in any statistical models. All model assumptions were met, and no data transformations were applied.

## 3 Results

Amino acid supplementation significantly explained variation in body mass between day 4 and day 8, but this effect was dependent on nestling age, initial (day 4) body mass, and habitat (four‐way interaction, *p* = 0.002, Table 1). Treatment effects were more pronounced in lighter nestlings and differed between forest and suburban habitats (Table 1; Appendix S1). Analysis of the random effects revealed significant differences both among broods (*σ* ^2^ = 0.11) and among nestlings within broods (*σ* ^2^ = 0.028). The treatment did not affect tarsus and wing length, either alone or in interaction with another variable (Appendix S2A,B). Tarsus and wing length were only explained by initial (day 4) body mass and the interaction between habitat and age (Appendix S2A,B), indicating that nestlings grew their tarsus and wing at a higher rate in forest than in suburban habitat. In contrast to body mass, individual identity did not explain significant variation in either tarsus or wing length (Appendix S2A,B).

**TABLE 1:** ANOVA results for the effects of treatment, habitat type, initial body mass, and nestling age on great tit nestling raw body mass measurement taken between days 4 and 8 post‐hatching.

Variable	Num df	Den df	*F*	*p*
Treatment	2	756.04	2.03	0.132
Habitat	1	777.53	3.07	0.080
**Initial body mass**	1	806.93	210.49	**< 0.001**
**Nestlings age**	1	692.63	491.29	**< 0.001**
Habitat: initial body mass	1	806.93	2.87	0.091
**Habitat: nestlings age**	1	692.63	7.02	**0.008**
Initial body mass: nestlings' age	1	687.92	3.78	0.052
**Habitat: treatment**	2	756.04	3.23	**0.040**
Initial body mass: treatment	2	756.65	2.15	0.117
Age: treatment	2	702.47	2.79	0.062
**Habitat: initial body mass: nestlings age**	1	687.92	5.25	**0.022**
**Habitat: initial body mass: treatment**	2	756.65	3.53	**0.030**
**Habitat: nestlings age: treatment**	2	702.47	5.86	**0.003**
**Initial body mass: nestlings' age: treatment**	2	697.65	3.26	**0.039**
**Habitat: initial body mass: nestlings age: treatment**	2	697.65	6.30	**0.002**

*Note:* Nestlings received 100 μL of either methionine solution, leucine solution, or tap water (control group) through oral supplementation. Columns display degrees of freedom (numerator and denominator), *F*‐values, and associated *p*‐values. Statistically significant effects (*p‐*value < 0.05) are indicated in **bold**.

These results were corroborated by growth rate (which was calculated from BLUP slope estimates) analyses, revealing that the effects of amino acid supplementation were strongly context‐dependent, emerging through a significant three‐way interaction between treatment, initial body mass, and habitat (Table 2). Although there was no overall effect of treatment on growth rates, both methionine and leucine supplementation significantly increased growth rates in smaller nestlings (1.45 ± 0.05 g/day, mean ± SE), but this benefit was restricted to the forest habitat (Figure 1). In contrast, larger forest nestlings showed reduced growth when supplemented (0.09 ± 0.03 g/day, mean ± SE), suggesting a possible cost of excessive amino acid intake in well‐developed individuals. These treatment effects were absent in the suburban habitat, where growth patterns differed: small suburban nestlings tended to grow faster than larger ones, but did not benefit from supplementation (Figure 1; Appendix S3).

**TABLE 2:** ANOVA results for fixed effects on great tit nestling body mass growth rate (BLUP slope estimates).

Variables	Num df	Den df	*F*	*p*
Treatment	2	138.97	1.42	0.244
**Habitat**	1	141.48	4.28	**0.040**
**Initial body mass**	1	156.62	16.02	**< 0.001**
Habitat: Initial body mass	1	156.62	3.66	0.057
**Habitat: treatment**	2	138.97	3.06	**0.050**
Initial body mass: treatment	2	139.03	1.90	0.154
**Habitat: initial body mass: treatment**	2	139.03	3.27	**0.041**

*Note:* Nestlings were randomly grouped into three treatment groups and received either methionine, leucine, or tap water (control group) orally from the age of day 4 to 7. Columns show degrees of freedom (numerator and denominator), *F*‐values, and associated *p*‐values. Significant effects (*p* < 0.05) are shown in **bold**.

**FIGURE 2:**
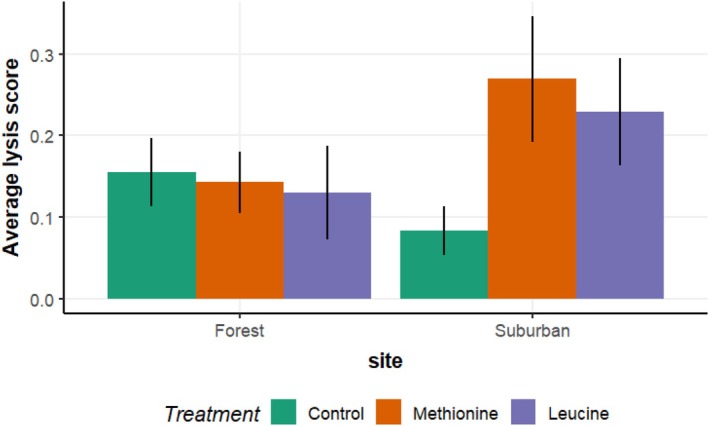
Lysis (titer) of forest and suburban great tit nestlings supplemented with methionine, leucine, or tap water from age of day 4 to 7 post‐hatching.

Agglutination (reflecting natural antibody titers) was not affected by amino acid supplementation (*F*_2,144.3 =_ 0.827, *p* = 0.439), habitat (*F*_1,112.1_ = 0.23, *p* = 0.629), initial body mass (*F*_2,124_ = 0.975, *p* = 0.325), or any of the interactions among these variables. In contrast, the effect of supplementation on lysis (reflecting complement activity) was dependent on habitat type and initial body mass. Lysis was lower in forest nestlings compared to suburban ones (*z* = −2.37, *p* = 0.017), albeit this difference got smaller in initially larger nestlings (*z* = 2.00, *p* = 0.044). Moreover, although none of the amino acid treatments affected lysis in the forest, nestlings in the suburbs treated with amino acids had higher lysis, although this only reached statistical significance in the case of methionine (methionine: *z* = 2.22, *p* = 0.026, leucine: *z* = 1.56, *p* = 0.118, Figure 2). However, post hoc pairwise comparisons among treatment groups within sites did not show any significant differences (all *p* > 0.05).

## 4 Discussion

Our study demonstrates that supplementation with the essential amino acids methionine and leucine affects nestling growth and aspects of baseline innate immune function in ways that depend on both habitat and individual condition. In forest habitat, both amino acids enhanced growth in initially smaller nestlings but reduced it in initially larger ones, suggesting a response that depends on the growth trajectory. Conversely, in the suburban habitat, supplementation did not significantly affect growth regardless of nestling size. When examining two parameters of innate baseline immune function, methionine‐supplemented suburban nestlings exhibited improved complement activity (measured as lysis), whereas no such effect was observed in forest habitat nestlings. As a result, the effects of amino acid supplementation on these traits appeared to be condition‐ and context‐specific.

Similar to our findings in suburban nestlings, previous studies have shown that methionine enhances immune responses in great tit nestlings (Brommer 2004; Tschirren et al. 2005). Also, Soler et al. (2003) observed the same pattern in methionine‐supplemented magpie nestlings. However, Wegmann et al. (2015) found no effect of methionine on growth rates in great tit nestlings, which partially contradicts our findings. In blue tits, methionine improved immune responses but only in parasitized nests, where nestlings with experimentally enhanced immunocompetence grew faster (Pitala et al. 2010). In Japanese quail, embryonic supplementation of methionine and leucine had programmatic effects on postnatal growth, whereas nutritional treatment with the same amino acids after hatching had no detectable effect on growth (Ndunguru et al. 2024a, 2024b, Reda et al. 2026, in prep). Additionally, methionine combined with choline significantly enhanced antibody titers in broilers (Swain and Johri 2010). However, short‐term immune benefits may not always translate into long‐term advantages, as nestlings often compensate for initial growth reductions and achieve comparable body sizes before fledging (Senécal et al. 2021). Collectively, these findings demonstrate the context‐dependent and tightly regulated allocation of resources between growth and immune defense, shaped by environmental pressures and survival costs (Brommer 2004; Pitala et al. 2010).

Suburban nestlings showed higher complement activity following the supplementation compared to their forest counterparts. This difference suggests that suburban environments may impose greater nutritional limitations, making nestlings more responsive to dietary improvements. Although urbanization is often associated with suppressed immune performance such as lower haptoglobin levels and greater body mass loss in urban great tit nestlings (Bailly et al. 2016), as well as reduced lysis linked to exposure to pollutants like lead, arsenic, and cadmium (Vermeulen et al. 2015), elevated lysis responses have also been reported in urban contexts, including in urban‐breeding black sparrowhawk nestlings (Nwaogu et al. 2023). Together, these findings indicate that urbanization can create developmental constraints through poor nutritional conditions such as limited access to high‐quality prey (e.g., caterpillars) and exposure to pollutants like arsenic from anthropogenic waste (Bailly et al. 2016; Biard et al. 2017; Sánchez‐Virosta et al. 2018; Senar et al. 2021). Additional environmental and anthropogenic stressors and human disturbance may further compromise nestling development in urbanized habitats (Demeyrier et al. 2017; Kasprzykowski et al. 2014), contributing to reduced growth (Maness and Anderson 2013) and potentially shortened lifespan (Salmón et al. 2016).

In general, amino acid supplementation can enhance growth in underdeveloped individuals under natural conditions, but may carry risks in others depending on habitat context and body condition. Nestlings raised in higher‐quality habitats tend to grow faster and exhibit better individual fitness (McKinnon et al. 2012; Stantial et al. 2021), whereas poor habitat quality has been associated with reduced reproductive success (Saulnier et al. 2022; Seress et al. 2020; Wawrzyniak et al. 2020). Structurally complex forest habitats are known to support better nutritional status in great tits (Seress and Liker 2015), as reflected in wider feather growth bars (Catfolis et al. 2023). Also, urbanization has been linked to significant genetic differentiation between urban and forest populations of great and blue tits, potentially altering their development and reproductive outcomes (Bisikirskienė et al. 2024). A recent review also emphasizes how urban‐driven shifts in vegetation, resource availability, and abiotic stressors, including light and noise pollution, can influence breeding and development in insectivorous birds such as great tits (Chen et al. 2023).

Our findings suggest that early postnatal nutrition can modulate growth trajectories in a context‐ and condition‐dependent manner. Nestlings with lower initial body mass (i.e., lighter nestlings) showed slower growth rates compared to their heavier counterparts, especially in the forest habitat, but this disadvantage was partially mitigated by amino acid supplementation. In particular, both methionine and leucine enhanced the growth of small nestlings in the forest, indicating a compensatory effect under protein‐limited conditions. Interestingly, these same treatments had no effect on suburban nestlings and even appeared to reduce growth in heavier forest nestlings, suggesting that amino acid availability interacts with individual condition and ecological context. Access to methionine and leucine likely depends on the availability of high‐quality arthropod prey, such as caterpillars and spiders, which are known to be less abundant or more variable in suburban and urban habitats (e.g., Seress et al. 2020). Reduced arthropod availability may therefore constrain the natural intake of essential amino acids in suburban environments, helping to explain habitat‐specific growth responses. Lighter nestlings may be particularly vulnerable to limitations because of competitive disadvantages within broods, making them more sensitive to variation in dietary amino acid availability. This supports the idea that nutrient supplementation can alleviate developmental constraints in poorly conditioned individuals, but excess amino acids may become unnecessary (or even costly) when nutritional needs are already met.

Initial body mass emerged as a strong predictor of growth trajectories in our study, and this is in line with a previous study that identified it as a key determinant of development and survival outcomes in birds (O'Connor 1975; Ronget et al. 2018). Although we did not track post‐treatment survival, which remains beyond the scope of this study, future work could explore how supplementation affects longer‐term fitness outcomes. Variation in initial body mass likely reflects differences in pre‐hatching maternal investment, particularly egg size (Krist 2011; Schifferli 1973). Nestling growth rate is a key determinant of post‐fledging survival, as the biggest and heaviest individuals at fledgling show higher survival. Traits such as body mass index, wing length, and hatching date have been shown to be reliable predictors of survival in altricial birds (Aastrup et al. 2023; Maness and Anderson 2013). Immune traits can also provide predictive power: in laying hens, higher titers of natural antibodies are indicative of increased survival (Sun et al. 2011). In our study, body mass and lysis activity were strongly influenced by habitat type and nestlings' initial (day 4) body mass, whereas tarsus and wing length were explained by age and initial body mass alone.

In conclusion, amino acid supplementation can enhance nestlings' development, but its effectiveness is based on the individual's initial body condition and ecological context. Methionine supplementation improved innate immune function (complement activity) in suburban nestlings, whereas no such effect was observed in forest environments. Furthermore, smaller nestlings in the forest habitat exhibited compensatory growth when provided with methionine or leucine supplementation. Future research should explore the effect of varying concentrations of leucine, methionine, and other amino acids (such as taurine) (Arnold et al. 2007; Ramsay and Houston 2003) and investigate long‐term consequences of amino acid supplementation during early age on future survival and reproduction under different ecological conditions.

## Author Contributions

**Ashetu Debelo Terefa:** conceptualization (equal), formal analysis (equal), investigation (equal), methodology (equal), writing – original draft (lead), writing – review and editing (equal). **Arne Hegemann:** methodology (equal), resources (equal), validation (equal), writing – review and editing (equal). **Zoltán Németh:** conceptualization (equal), funding acquisition (equal), investigation (equal), methodology (equal), project administration (equal), resources (equal), supervision (equal), validation (equal), writing – review and editing (equal). **Ádám Z. Lendvai:** conceptualization (equal), data curation (lead), formal analysis (equal), funding acquisition (equal), investigation (lead), methodology (equal), project administration (equal), resources (equal), supervision (equal), validation (equal), writing – review and editing (equal).

## Funding

The study was funded by the National Development, Research and Innovation Fund (OTKA K139021 and ADVANCED 153291 to ÁZL). AD received a Stipendium Hungaricum Scholarship from Tempus Public Foundation for Ph.D. studies. We acknowledge support from the University of Debrecen Program for Scientific Publication. AH was funded by a grant from the Swedish Research Council (2024‐05731).

## Conflicts of Interest

The authors declare no conflicts of interest.

## Supporting information


**Appendix S1:** ece373284‐sup‐0001‐Appendices.docx.

## Data Availability

All data used during the analysis in this manuscript are attached as Supporting Information.
