# Supplementary material for: Context‐Dependent Effects of Amino Acid Supplementation on Nestling Growth and Baseline Innate Immune Function
Source: Ecol Evol. 2026 Mar 18;16(3):e73284. doi: 10.1002/ece3.73284 (PMC13093782; doi:10.1002/ece3.73284)
Supplement: Supplementary file 1 — Appendix S1: ece373284‐sup‐0001‐Appendices.docx. [file ECE3-16-e73284-s001.docx]

**Appendices**

**Appendix 1.** Figure showing initial day body mass and subsequent mass measurements of great tit nestlings from forest and suburban habitats. Nestlings received oral supplementation of either methionine, leucine or tap water (control groups) from day 4 to 7 of age.


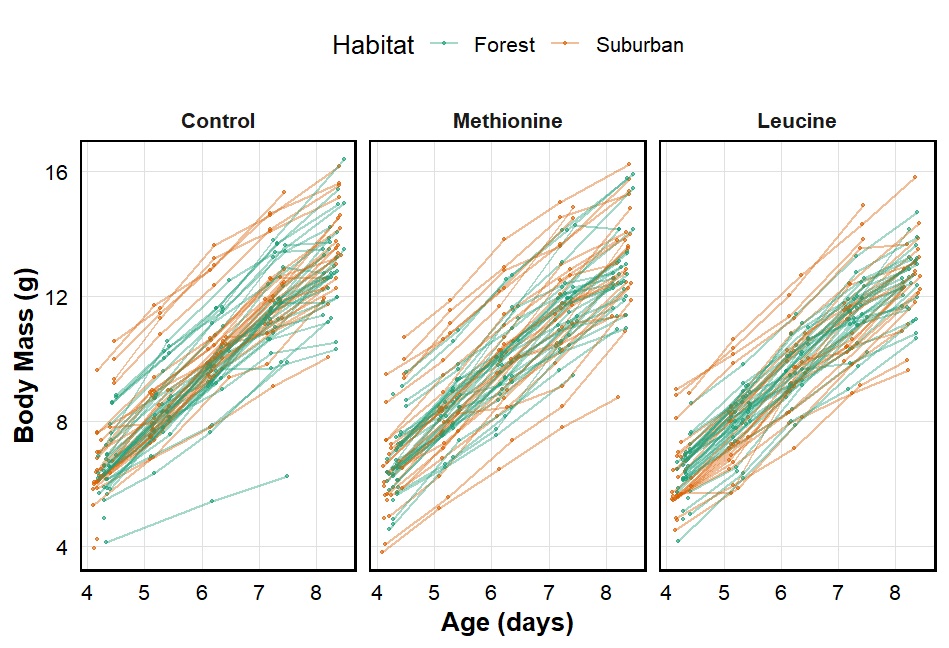


**Appendix 2**. ANOVA results of great tit nestlings wing and tarsus length analyses. Nestlings received methionine, leucine solution or tap water (control group) from day 4 to 7 of ages.

A) Wing length

| **Variables** | **NumDF** | **DenDF** | **F value** | **P-value** |
| --- | --- | --- | --- | --- |
| Treatment | 2 | 263.89 | 0.002 | 0.998 |
| **Initial body mass** | 1 | 183.66 | 97.58 | **< 0.001** |
| **Habitat** | 1 | 169.34 | 4.333 | **0.038** |
| **Nestlings age** | 1 | 274.153 | 3886.15 | **< 0.001** |
| **Habitat: age** | 1 | 274.06 | 4.86 | 0.02 |

B) Tarsus length

| **Variables** | **NumDF** | **DenDF** | **F value** | **P-value** |
| --- | --- | --- | --- | --- |
| Treatment | 2 | 144.17 | 0.27 | 0.77 |
| **Habitat** | 1 | 74.03 | 12.38 | **< 0.001** |
| **Initial body mass** | 1 | 242.37 | 115.76 | **< 0.001** |
| **Age** | 1 | 184.27 | 404.77 | **<0.001** |
| **Habitat: age** | 1 | 156.94 | 15.28 | **<0.001** |
| **Initial body mass: age** | 1 | 183.69 | 7.85 | **0.01** |

**Appendix 3**. Great tit nestlings body mass, tarsus length and wing length growth rate within forest and suburban habitats.

| **Habitat** | **Brood size category** | **Mean mass growth rate (g/day)** | **Mean Tarsus length growth rate (mm/day)** | **Mean wing length growth rate (mm/day)** |
| --- | --- | --- | --- | --- |
| Forest | Light | 1.45 ± 0.05 | 2.00 ± 0.03 | 3.81 ± 0.08 |
| Forest | Intermediate | 1.45 ± 0.04 | 1.95 ± 0.03 | 3.86 ± 0.13 |
| Forest | Heavy | 1.54 ± 0.03 | 1.91 ± 0.05 | 4.16 ± 0.11 |
| Suburban | Light | 1.52 ± 0.05 | 1.89 ± 0.05 | 3.34 ± 0.17 |
| Suburban | Intermediate | 1.60 ± 0.04 | 1.85 ± 0.05 | 3.62 ± 0.25 |
| Suburban | Heavy | 1.54 ± 0.03 | 1.75 ± 0.04 | 4.57 ± 0.09 |
